# Exploring the Ecological Impacts of Herbicides on Antibiotic Resistance Genes and Microbial Communities

**DOI:** 10.3390/life15040547

**Published:** 2025-03-26

**Authors:** Yunfei Zhao, Yixiao Wang, Jie Lu, Baoli Zhu, An-Dong Li

**Affiliations:** 1 School of Public Health, Nanjing Medical University, Nanjing 211166, China; panda_fei_11582@163.com (Y.Z.); yixiaowang200203@163.com (Y.W.); 2 Key Laboratory of Environmental Medicine Engineering of Ministry of Education, Southeast University, Nanjing 210009, China; kyirestrive1026@163.com; 3 Engineering Research Center of Health Emergency, Jiangsu Provincial Center for Disease Control and Prevention, Nanjing 210009, China; 4 Department of Occupational Disease Prevention, Jiangsu Provincial Center for Disease Control and Prevention, Nanjing 210009, China

**Keywords:** antibiotic resistance genes, herbicides, microbial community, metagenomics

## Abstract

The widespread application of herbicides has profound ecological consequences, particularly regarding the distribution of antibiotic resistance genes (ARGs) and microbial communities. In this study, we analyzed herbicide-related metagenomic data to assess the impact of herbicide exposure on ARGs and microbial populations. Our results demonstrate that herbicide application significantly increased the abundance of ARGs, particularly those associated with multidrug resistance, sulfonamides, and bacitracin, with notable increases in subtypes such as *bacA* and *sul1*. Microbial community analyses revealed a dominance of Pseudomonadota and Actinomycetota, along with a significant down-regulation of genera like *Fibrisoma*, *Gilsonvirus*, *Limnobacter*, and *Wilnyevirus* in the experimental group. Additionally, herbicide exposure led to a marked reduction in biodiversity. When threshold values were relaxed, correlation analyses revealed a co-occurrence pattern between multiple genes and *sul1*, suggesting that horizontal gene transfer plays a pivotal role in the spread of antibiotic resistance in herbicide-contaminated soils. Moreover, environmental factors were found to significantly influence both microbial community composition and ARG distribution. These findings highlight the complex ecological effects of herbicides on microbial diversity and the dissemination of resistance genes, emphasizing the need for further research into the long-term environmental and public health implications of herbicide use.

## 1. Introduction

Antibiotic resistance is a pressing global issue [1]. The widespread use of pesticides can significantly contribute to the accumulation of antibiotic resistance in the environment, thereby facilitating the spread of antibiotic-resistant bacteria [2]. Among the various types of pesticides, herbicides are the most widely used and produced worldwide, making them a particular area of concern. However, research on the biological hazards associated with herbicides, particularly in relation to antibiotic resistance in the environments where they are applied, remains insufficiently explored.

Previous studies have demonstrated that antibiotic resistance genes (ARGs) are disseminated in non-infectious disease processes, including in environments such as soil [3], oceans [4], sediments [5], and glaciers [6]. Many ARGs associated with clinical pathogens, such as *ctx*-m (mediating cephalosporin resistance) [7], qnr (mediating quinolone resistance) [8], and van (mediating vancomycin resistance) [9], have also been detected in these areas. This is likely due to the homology of ARGs carried by non-pathogenic bacterial species, highlighting the potential for antibiotic resistance exchange between human pathogens and non-pathogenic bacteria [10]. Agricultural land, serving as a critical interface between humans and the natural environment, is an area of increasing concern due to its role in ARG enrichment [11]. Herbicides may contribute to the accumulation of ARGs in these environments, potentially through mechanisms such as cross-resistance with antibiotics [12]. However, the specific distribution patterns and subsequent impacts of herbicides on ARG enrichment remain unclear.

Herbicide use has been shown to significantly increase the abundance of ARGs in soil and water samples from the environments where they are applied [13,14]. Comparative analyses of high-throughput sequencing data from environmental metagenomic samples against antibiotic resistance databases revealed that herbicides not only increase the abundance of ARGs but also substantially expand their diversity. Additionally, a large number of transposons and other horizontal gene transfer (HGT) elements were selected and enriched in these environments [15]. Research has also shown that ARGs associated with antibiotic efflux mechanisms and multidrug resistance are detected in large quantities in environmental samples exposed to herbicides [16]. Herbicides can induce gene mutations [17] and enhance the binding and transfer of ARGs by increasing cell membrane permeability and the content of mobile genetic elements (MGEs) in microorganisms, thereby facilitating the spread of ARGs in these environments [18]. Vertical gene transfer and HGT through MGEs are the two primary mechanisms for ARG propagation in the environment [19]. Furthermore, attention should be given to human pathogens carrying ARGs, as virulence factors enable these pathogens to invade human hosts and cause disease [20].

We hypothesize that gene mutations induced by herbicide stress may enable host microorganisms to acquire ARGs. Simultaneously, the accumulation of numerous HGT elements accelerates the spread of ARGs in environments where herbicides are applied, thereby increasing the potential in vivo harm caused by pathogenic bacteria. However, it remains unclear whether the enrichment of ARGs due to herbicides follows similar patterns across samples from different regions. Additionally, it is uncertain whether the taxonomic profile determines the ARG profile. To address these questions, we employed metagenomic analyses techniques on datasets from different regions to explore the relationships between taxonomic and ARG profiles.

## 2. Methods

### 2.1. Data Collection

After reviewing relevant research articles on herbicide contamination, a total of 185 datasets were collected, including 88 water samples and 97 soil samples. These metagenomic datasets were obtained from the NCBI Sequence Read Archive (SRA) and were selected based on specific inclusion criteria to ensure consistency and comparability across samples. First, only datasets generated using the Illumina high-throughput sequencing platform were included to maintain uniform read length and quality metrics while minimizing technical variability. Second, each dataset was explicitly linked to herbicide-contaminated environments, with metadata confirming the presence of herbicide pollution. Public databases were systematically screened to identify relevant studies, and datasets from studies lacking essential metadata or exhibiting low sequencing depth were excluded. Additionally, a standardized bioinformatics pipeline was applied uniformly to all samples to reduce potential biases during data processing. All sequencing data were downloaded and converted to FASTQ format using the SRA Toolkit (v3.0.0). A comprehensive summary of all datasets is provided in Table S1.

### 2.2. ARG Profile

In this study, we employed a similarity-based search approach to annotate and classify ARG-like sequences in the metagenomic data. Firstly, sequence alignment was performed using BWA with default parameters (E-value cutoff for GreenGenes: 1 × 10^−10^; E-value cutoff for Essential Single Copy Marker Genes: 3; Identity cutoff for Essential Single Copy Marker Genes: 45%; Query cover cutoff for Essential Single Copy Marker Genes: 0%). Subsequently, functional annotation was conducted using BLASTX, again with default parameters (E-value cutoff for target sequences: 1 × 10^−7^; Identity cutoff for target sequences: 80%; Query cover cutoff for target sequences: 75%; Aligned length cutoff (in amino acids) for target sequences: 25%) [21].

### 2.3. Taxonomic Assignment

To investigate the composition of microbial communities, Kraken 2, a taxonomic classification system, was employed. This system utilizes precise k-mer matching to achieve high accuracy and rapid classification. In this study, Kraken 2 was used with confidence as 0 and minimum-hit-groups as 2 [22]. The database for microbial species annotation was the standard database provided with Kraken 2. Additionally, multiple Kraken reports were combined into a single report for subsequent analyses.

### 2.4. Species Diversity Analysis

Alpha and beta diversity analyses are described in detail in this study. We selected Shannon index and richness as metrics for assessing alpha diversity. To further visualize differences among samples, principal coordinates analysis (PCoA) was performed using distance metrics. If two samples are positioned closely on the PCoA plot, their species compositions are considered similar. PCoA was conducted using the R software package (v4.3.3), with beta diversity distance matrices calculated by the vegan package (v2.6-8).

### 2.5. Network Analysis

To investigate the co-occurrence patterns between microbial communities and resistomes, a correlation matrix was constructed by calculating all possible pairwise Spearman’s rank correlations between the 1489 ARG subtypes and 4571 bacterial genera detected across 185 samples in R environment. The resulting correlation matrix was then converted into an association network using the Gephi software (v0.10.1), focusing on samples with a detection rate of over 90% for non-zero values. A correlation between two items was considered statistically robust if the Spearman’s correlation coefficient (ρ) was greater than 0.9 and the *p*-value was less than 0.001.

### 2.6. Statistical Analys

Statistical tests were conducted to determine whether significant differences existed between the groups. Prior to performing the tests, a normality check was conducted to determine the type of data distribution. ANOVA was applied when the data met the assumption of normality, while the Kolmogorov–Smirnov test was used when this assumption was violated. All statistical analyses were performed using SPSS version 27.0.1.

## 3. Results

### 3.1. Impact of Herbicides on ARGs Abundance in Different Regions

Metagenomic data from soil and water samples contaminated with herbicides were collected from various regions from public databases. The datasets include soil samples from multiple locations, such as Qingdao (S_Qingdao), Nanjing (S_Nanjing), Haikou (S_Haikou), Mianyang (S_Mianyang), and Yingtan (S_Yingtan) in China, as well as Diamantina (S_Diamantina) in Brazil. Control soil samples, free from herbicide contamination, were also collected from Qingdao and Haikou (CK_Qingdao, CK_Haikou). Additionally, water samples were obtained from Mont-Saint-Hilaire (W_Mont-Saint-Hilaire) in Canada (Table 1). For comparison, control soil samples, identified as free from herbicide contamination, were collected from Qingdao (CK_Qingdao) and Haikou (CK_Haikou). The classification of herbicide-contaminated and control samples was based on metadata from publicly available databases and descriptions in the original source publications [23,24]. According to these sources, control samples were obtained from pristine, untreated environments, ensuring that they were not exposed to herbicide contamination (Table S2).

To assess the impact of herbicide contamination on the abundance of ARGs, we analyzed the ARG abundance in the collected datasets. The results revealed that the total abundance of ARGs was lower in the water samples from Mont-Saint-Hilaire (W_Mont-Saint-Hilaire) compared to the soil contamination group. Additionally, the mean abundances of *novA* (novobiocin), *bacA* (bacitracin), *sul1* (sulfonamide), and *MexF* (multidrug) were elevated in the soil samples (Figure S1). In Haikou, the abundance of ARGs was higher in the contaminated samples compared to the control group, with a notable increase in *bacA* in the Haikou samples (Figure S1) (Kolmogorov–Smirnov test, *p*-value < 0.001). This increase may be attributed to the significant effect of herbicide contamination on the abundance of ARGs. Overall, Figure S1 illustrates that *bacA* exhibited the highest abundance in S_Mianyang, S_Haikou, and W_Mont-Saint-Hilaire, whereas *novA* was most abundant in S_Yingtan and S_Diamantina.

### 3.2. Impact of Herbicides on the Distribution of ARGs in Different Regions

**Figure 1:**
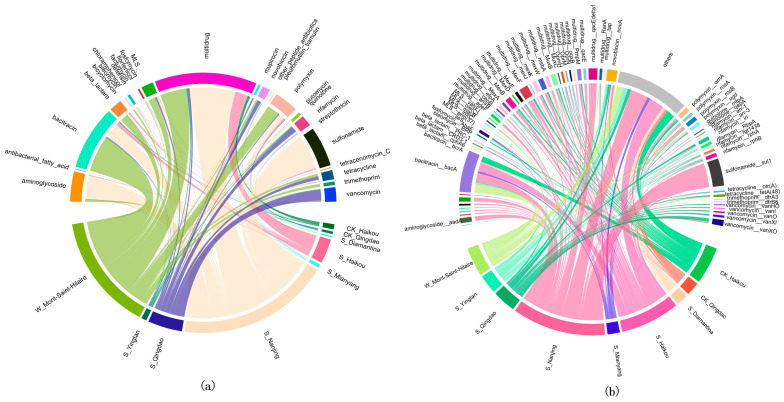
(**a**) Circos analysis of ARGs across different groups (S_Nanjing, S_Yingtan, S_Mianyang, S_Haikou, S_Qingdao, S_Diamantina, W_Mont-Saint-Hilaire, CK_Qingdao, CK_Haikou), categorized by broad antibiotic classes. (**b**) Circos analysis of ARG subtypes across the same groups.

To enhance the visualization of ARG distribution across different groups, a Circos analysis was performed, which identified 27 distinct ARG types across the nine groups (Figure 1a). Multidrug resistance genes and bacitracin resistance genes were more abundant and widely distributed compared to other ARG types in these samples. Additionally, the heatmap indicates that multidrug resistance genes were most abundant in the Haikou region (Figure S2). Furthermore, the abundance of bacitracin resistance genes was higher in W_Mont-Saint-Hilaire, while sulfonamide resistance genes were more abundant in S_Nanjing. Meanwhile, resistance genes exhibit higher abundance in water samples compared to the majority of soil samples (Figure 1a). Moreover, the top 20 ARG subtypes by abundance were selected for each group, resulting in a total of 71 distinct ARG subtypes. As shown in Figure 1b, *bacA* (bacitracin) had the highest detection rate, followed by *sul1* (sulfonamide), *novA* (novobiocin), and others. Meanwhile, the detection rate of *sul1* was relatively high in S_Nanjing, whereas the average abundance of *bacA* remained consistently high in W_Mont-Saint-Hilaire, S-Haikou, and CK-Haikou. Notably, the S_Nanjing samples exhibited greater diversity in both resistance gene types and subtypes, with a higher richness of resistance genes compared to other soil groups (Figure 1a,b).

**Figure 2:**
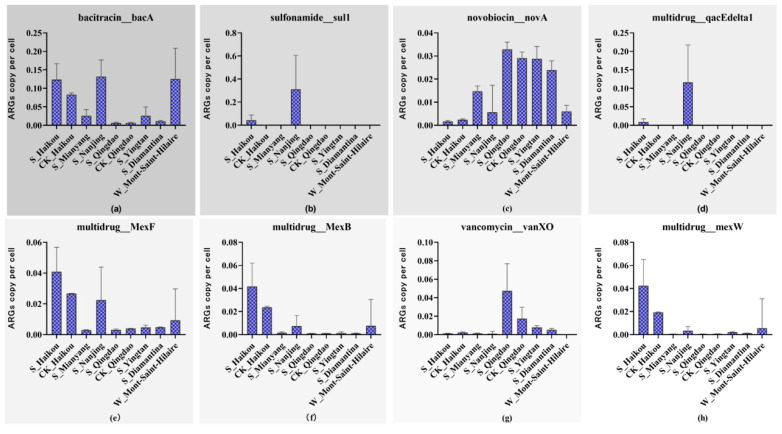
Abundance of ARGs in S_Nanjing, S_Yingtan, S_Mianyang, S_Haikou, S_Qingdao, S_Diamantina, W_Mont-Saint-Hilaire, CK_Qingdao, and CK_Haikou groups; (**a**) *bacA* (bacitracin), (**b**) *sul1* (sulfonamide), (**c**) *novA* (novobiocin), (**d**) *qacEdel-ta1* (multidrug), (**e**) *MexF* (multidrug), (**f**) *MexB* (multidrug), (**g**) *vanXO* (vancomycin), (**h**) *mexW* (multidrug). Grayscale of the background represents the total abundance value of the ARGs, with darker grayscales representing higher abundance values.

Furthermore, as shown in Figure 2, *bacA* was the most abundant among the top 8 resistance genes, particularly in the contaminated groups, with S_Nanjing and W_Mont-Saint-Hilaire exhibiting the highest abundances. Meanwhile, *novA* exhibited relatively high abundance across all groups, with particularly elevated levels in S_Qingdao, CK_Qingdao, S_Yingtan, and S_Diamantina. Figure 2 also indicates that the abundance of ARGs varies across different regions, with certain genes exhibiting higher prevalence in specific locations. For example, the abundance of *MexB* (multidrug) and *MexW* (multidrug) was higher in the Haikou samples, including both contaminated and control groups, compared to those from other regions. Likewise, *vanXO* (vancomycin) exhibited the highest abundance in the Qingdao samples, while *sul1* and *qacEdel-ta1* (multidrug) were most abundant in S_Nanjing (Figure 2).

The Venn diagram illustrates that S_Nanjing exhibited the highest total number of ARGs (620 genes), while S_Diamantina had the lowest (232 genes). The distribution of ARGs across the contaminated soil groups identified a total of 130 shared ARGs, highlighting a core set of ARGs in herbicide-contaminated soil environments, with *bacA*, *novA*, and *MexF* being the most abundant (Figures S6A and S3). Each group also exhibited a unique ARG profile. The highest number of unique ARG subtypes was observed in S_Nanjing (157), followed by S_Haikou (91) and S_Qingdao (65) (Figure S6A). In contrast, S_Diamantina exhibited the fewest unique ARG subtypes, with only four genes. As coastal cities, S_Haikou and S_Qingdao showed a higher degree of shared ARGs between these two groups (Figure S6). A comparison of the experimental and control soil groups from Qingdao revealed 236 shared ARGs and 240 unique genes, while in Haikou, 276 shared genes and 194 unique genes were identified (Figure S6B,C).

**Figure 3:**
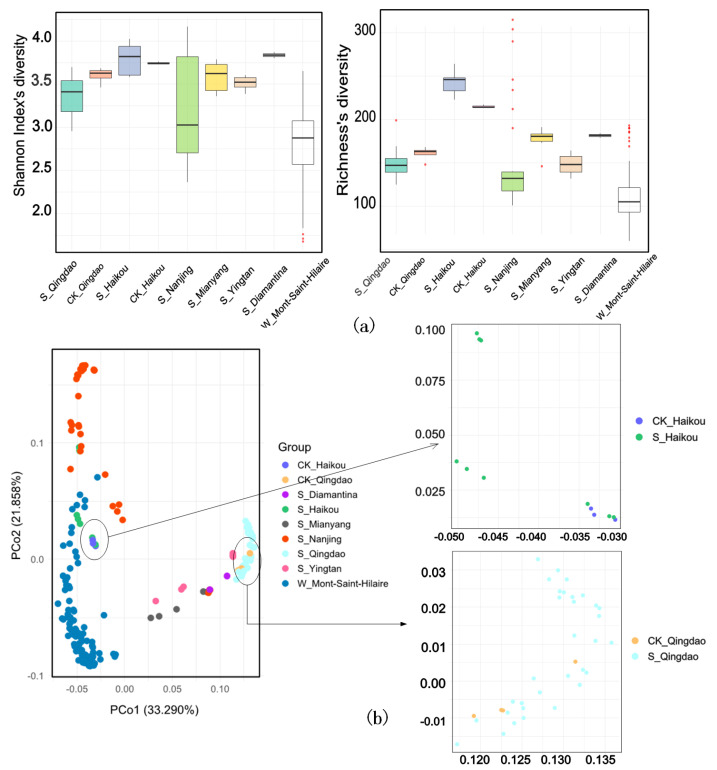
(**a**) Alpha diversity indices boxplot in different groups. (**b**) PCoA based on ARGs abundance between groups. X-axis, first principal component and Y-axis, second principal component; 33.290% in brackets represents contributions of PC1 components to samples, 21.858% represents contributions of PC2 components to samples. A dot represents each sample, and different colors represent different groups.

To assess the diversity of ARGs in each group, we evaluated the samples using both the Shannon index and richness. Additionally, we observed that the control group in the Qingdao samples exhibited higher biodiversity than the contaminated group, whereas the Haikou samples showed the opposite trend. Meanwhile, we performed linear regression analysis to examine the relationship between the alpha diversity of both microbial communities and ARGs with total ARG abundance (Figures S9 and S10). The results showed a significant positive correlation between microbial alpha diversity (Shannon index and Richness) and total abundance. Further analyses of samples from Qingdao and Haikou confirmed this positive correlation, while ARG richness also exhibited a significant correlation with total ARG abundance (Figure S9). Furthermore, both the Shannon index and richness were lowest in W_Mont-Saint-Hilaire, wherein principal coordinates analysis (PCoA) based on Bray–Curtis distance metrics was notably distant from the other groups, forming a distinct cluster (Figure 3a,b). This clustering could be attributed to unique environmental factors, such as geographic location or habitat characteristics. Similarly, S_Nanjing also clustered separately from other soil samples and exhibited lower Shannon index and richness values (Figure 3a,b).

### 3.3. Impact of Herbicides on Microbial Communities in Different Regions

**Figure 4:**
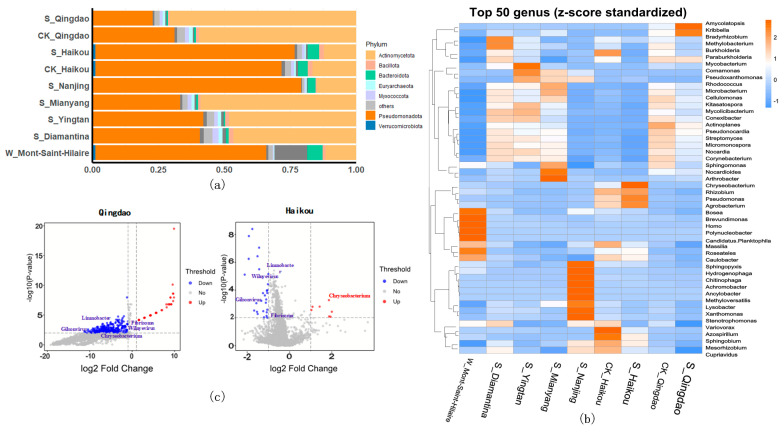
(**a**) Relative abundance of top 10 bacterial communities at phylum level. (**b**) Heatmap of average abundance of bacterial communities at genus level. (**c**) Volcano plots of differential expression of bacterial communities at genus level in Qingdao and Haikou groups.

Among all groups, the top 10 bacterial phyla by relative abundance were dominated by Pseudomonadota (22.63–79.06%) and Actinomycetota (11.50–70.63%) (Figure 4a and Figure S4). As shown in Figure 4a, Pseudomonadota and Actinomycetota exhibited higher abundances in both soil and aquatic environments. In the Haikou region, the proportion of Pseudomonadota in the polluted group (75.62%) was higher than in the control group (70.48%), while the proportion of Actinomycetota in the experimental group (12.21%) was lower than in the control group (16.47%) (Figure 4a and Figure S4). However, a contrasting trend was observed in Qingdao. The heatmap analysis revealed notable variations in bacterial abundance across different sites. *Pseudomonas* was more abundant in S_Haikou, *Amycolatopsis* in S_Qingdao, and *Bradyrhizobium* in S_Diamantina, in comparison to other sites (Figure 4b). Additionally, the bacterial communities in S_Nanjing were predominantly composed of *Ancylobacter* (15.52%), *Achromobacter* (7.60%), *Methyloversatilis* (5.54%), *Hydrogenophaga* (3.92%), *Chitinophaga* (2.89%), and other genera (Figure 4b and Figure S5A). In contrast, the bacterial communities in W_Mont-Saint-Hilaire were primarily dominated by *Homo* (13.16%), *Polynucleobacter* (7.91%), *Candidatus Planktophila* (3.54%), and other genera (Figure 4b and Figure S5B). In the Haikou contaminated and control groups, 7 genes were up-regulated and 27 genes were down-regulated, while in the Qingdao groups, 39 genes were up-regulated and 401 genes were down-regulated (Figure 4c). Specifically, compared to the control group, *Fibrisoma*, *Gilsonvirus*, *Limnobacter*, and *Wilnyevirus* exhibited down-regulation, while *Chryseobacterium* was up-regulated in Haikou but down-regulated in Qingdao (Figure 4c). Additionally, in the Qingdao region, both the Shannon and richness indices were lower in the contaminated groups (Figure S7A), compared to the control group (Kolmogorov–Smirnov test, *p*-value = 0.004 for Shannon and *p*-value = 0.013 for richness). Figure S7A also illustrates reduced biodiversity in the S_Nanjing and W_Mont-Saint-Hilaire groups. The results of PCoA at the genus level further confirmed that these two groups formed distinct clusters, clearly separated from the other samples (Figure S7B). Furthermore, a significant difference in distribution was observed between the contaminated and control groups (Figure S7B), indicating that herbicide contamination has a substantial impact on microbial communities (Kolmogorov–Smirnov test, *p*-value < 0.001).

### 3.4. Correlation Analysis Between ARGs and Microbial Communities Across Different Regions

**Figure 5:**
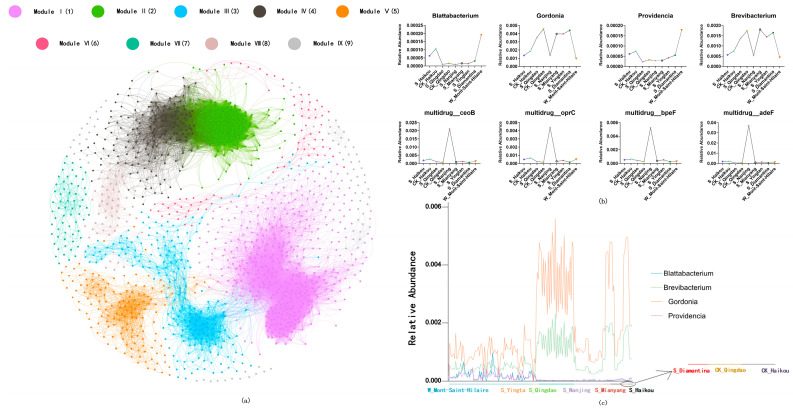
(**a**) Network analysis showing co-occurrence patterns between ARG types and microbial taxa across all treatments. Nodes with different colors represent different modularity classes, and edges correspond to strong (ρ > 0.9) and significant (*p* < 0.05) correlations between nodes. (**b**) Line plots of distribution of abundance of genus and ARGs in each group. (**c**) Line plots of distribution of abundance of genus in different samples.

The network analysis was performed to investigate the co-occurrence patterns between ARG subtypes and microbial taxa (at the genus level) across all treatments, based on strong (ρ > 0.9) and significant (*p* < 0.05) correlations (Figure 5a). The network results were divided into nine major modules based on modularity, with the four largest modules—Modules I, II, III, and IV—occupying 1158 of the total 1015 vertices. *Blattabacterium* was the central node in Module I, while *Gordonia* served as the central hub in Module II. *Providencia* functioned as the central node in Module III, and *Brevibacterium* was the core hub in Module IV. With the parameters we have set, no ARG subtypes had significant correlation with any microbial taxa. However, a significant correlation was observed among the four ARGs: *ceo*B (multidrug), *opr*C (multidrug), *bpe*F (multidrug), and *ade*F (multidrug). These genes exhibited similar distribution patterns across groups, with higher abundances in the S_Nanjing group compared to others. Notably, *ceo*B had the highest abundance across all groups (Figure 5b). Furthermore, *Gordonia* was consistently more abundant across all samples (Figure 5c).

## 4. Discussion

Numerous studies have demonstrated that herbicides significantly affect biodiversity, with the alpha diversity of microbial communities serving as a key indicator of ecosystem health [25,26,27]. This diversity reflects not only the abundance of microorganisms within the environment but also their essential ecological functions [28]. In recent years, herbicides, as widely used agricultural chemicals, have garnered significant attention due to their potential impacts on microbial communities and the distribution of ARGs [29]. In this study, we examined the variations in α-diversity across different contamination groups and found that microbial diversity was significantly higher in the control group than in the contaminated group for the Qingdao samples (Figure 3a and Figure S7A). This reduction in diversity in the contaminated group is likely attributed to the herbicide’s toxic effects, which impair microbial diversity [30]. Analyses of the data from the referenced study indicate that as herbicide concentrations increase, the variability among samples within the contaminated group becomes more pronounced [14]. This suggests that herbicide application disrupts microbial community composition and ARGs distribution, exacerbating community imbalances and intensifying selective pressure on microorganisms [26]. In contrast to the Qingdao samples, the Haikou samples exhibited an opposite trend in ARGs diversity (Figure 3a). This difference may be attributed to variations in herbicide types and regional environmental factors [31,32]. The increased ARG diversity observed in the Haikou samples may be linked to glyphosate contamination, which could provide a competitive advantage to certain ARGs with strong glyphosate tolerance, thereby influencing the ARG composition [13,33]. The Shannon index showed no significant difference (*p* > 0.05, *t*-test), while richness was significantly higher in the contaminated group (*p* = 0.0048, *t*-test), suggesting contamination promoted certain low-abundance ARGs without altering overall ARG evenness. The linear regression analysis revealed a significant correlation between ARG abundance and microbial diversity, implying herbicide contamination shapes ARG composition and distribution (Figures S9 and S10). However, PCoA results showed no significant differences in overall ARG composition between contaminated and control groups, suggesting limited impact on ARG structural composition (Figure 3b). These findings suggest that while herbicide contamination can influence ARG abundance and diversity, its effects on overall ARG composition remain inconclusive and may be influenced by other environmental factors [34]. Moreover, the greater dispersion of data points within the contaminated group suggests increased intra-group variability, implying that contamination may exert selective pressure on specific ARGs, thereby increasing compositional heterogeneity among samples [13]. This phenomenon may be associated with adaptive changes in certain ARGs under contamination stress [15], while the limited sample size of the Haikou control group may also have contributed to statistical fluctuations or biases in the results.

Both the Alpha diversity and PCoA results revealed significant shifts in microbial community composition, suggesting that sample characteristics play a dominant role in shaping microbial diversity. However, the increased abundance of ARGs in the contaminated group underscores the potential ecological consequences of herbicide application (Figure S1). Specifically, the addition of herbicides led to a notable increase in ARGs abundance, aligning with findings from a previous study [15]. The emergence of antibiotic resistance in microbes through the acquisition of ARGs occurs via several known pathways: de novo bacterial mutations, recombination, and HGT [35]. Recently, increasing evidence has shown that herbicide can facilitate the spread of ARGs [14,36]. Potential molecular mechanisms include enhanced intercellular contact via pilus-encoded gene expression, reduced cell surface charge, increased membrane permeability, and the promotion of DNA uptake through the proton motive force [17]. In the contaminated soil group, we found that the average abundance of multidrug, sulfonamide, and bacitracin resistance genes was significantly higher than that of other ARGs (Figure S2). This is likely influenced by environmental factors, such as agricultural practices and livestock husbandry [37,38]. The prevalence of multidrug resistance genes in contaminated soils likely reflects their adaptive advantage in environments with multiple chemical stressors [39]. These genes often encode efflux pumps or other mechanisms that confer resistance to a broad range of compounds, allowing bacteria to thrive in highly polluted conditions [40]. Moreover, novobiocin’s mechanism of action involves synergy with polymyxin to inhibit DNA gyrase, and it binds to and stimulates LptB, an ATPase responsible for lipopolysaccharide transport [41]. Given its cytotoxic properties [42], novobiocin is likely to inhibit the growth of competing microorganisms, including herbicide-sensitive strains [43], thereby creating a selective environment that favors the survival and proliferation of novobiocin-resistant or -tolerant microorganisms. This selective pressure may ultimately lead to an increase in the abundance of these resistant microorganisms within the soil.

Further analyses revealed significant variation in the average abundance of different ARG subtypes across the 185 samples, with *bacA*, *sul1*, and *novA* exhibiting markedly higher abundance compared to other isoforms (Figure 2). The ARG subtype *bacA* is commonly found in the natural environment, with its homologues present in a wide range of bacterial genera [44]. This suggests that the elevated abundance of *bacA* observed in our study may be attributed to the gene’s high genetic content within the soil environment. One study has shown that *bacA* encodes undecaprenol kinase, and when this enzyme is overproduced, it can produce sufficient amounts of undecaprenol monophosphate from undecaprenol, thereby overcoming the effects of undecaprenol pyrophosphate sequestration, which results in bacitracin resistance [45]. Furthermore, *bacA* mutants exhibited reduced virulence in a mouse model and increased susceptibility to bacitracin [46]. Based on these observations, it can be inferred that the up-regulation of *bacA* in response to herbicide-induced toxic stress may help mitigate the harmful effects of herbicides [46]. In addition, previous studies have demonstrated that herbicide concentrations are positively correlated with *sul1* abundance [47], which aligns with the findings of our study. The *sul1* gene is typically located on a MGE, such as a plasmid or integron, facilitating HGT [48]. It encodes dihydropteroate synthase, the target of sulfonamide antibiotics, thereby enabling the rapid spread of resistance within bacterial populations [49]. Notably, the abundance of *sul1* in some Nanjing samples was higher than in other groups, potentially due to the experimental application of sulfonylurea herbicides [50] and the agricultural soil context, where previous studies have reported a high detection rate of *sul1* in human and livestock feces [51,52,53]. Meanwhile, the high abundance of *novA* observed in our study suggests its involvement in novobiocin transport [54], with increased *novA* abundance being common in soils with elevated novobiocin levels. Additionally, the volcano plot of resistance genes from both the control and contaminated groups revealed no genes exhibiting similar trends (Figure S8). This discrepancy may be due to differences in herbicide treatments applied across the groups [23,24]. Meanwhile, certain ARGs exhibited higher abundances in samples from Haikou and Qingdao compared to other groups, which may be attributed to environmental differences across regions [55].

In recent years, numerous studies have shown that herbicides significantly impact the composition and functional dynamics of microbial communities [56,57,58]. In light of this, we further investigated the effects of herbicide contamination on microbial populations in this study. These shifts in microbial characteristics arise from both direct and indirect effects of herbicides, which influence microbial function and survival via environmental or host-mediated pathways, depending on the herbicide’s mode of action [59]. For instance, glyphosate directly affects microbial survival by inhibiting the enzyme 5-enolpyruvylshikimate-3-phosphate synthase in the shikimate pathway, which is crucial for the production of essential amino acids in both plants and many microbes [60]. Other herbicides, such as ALS inhibitors, disrupt branched-chain amino acid biosynthesis, ACC inhibitors interfere with fatty acid synthesis, and glutamine inhibitors impact nitrogen metabolism [59]. In contrast, some herbicides do not directly target microbes but instead disrupt plant cellular metabolism, including processes like photosynthesis and plant hormone biosynthesis [57]. In the present study, volcano plots revealed a decrease in the abundance of *Fibrisoma*, *Gilsonvirus*, *Limnobacter*, and *Wilnyevirus* in both the control and herbicide-contaminated groups from the Haikou and Qingdao areas (Figure 5c), indicating selective pressure exerted by herbicides on these microbial taxa. *Fibrisoma* is commonly found in environments with high organic matter content [61]. Herbicides may disrupt the organic matter decomposition process or alter environmental conditions [62], limiting the available resources for *Fibrisoma*, thereby reducing its abundance. Similarly, viruses such as *Gilsonvirus* and *Wilnyevirus* are highly sensitive to environmental disturbances, including chemical stressors like herbicides [63]. Herbicides can impact the microbial hosts of these viruses, potentially reducing the availability of suitable hosts for viral replication [64]. Meanwhile, *Limnobacter*, as a representative nitrifying bacterium crucial for nitrogen cycling in soil environments, faces significant inhibitory effects from herbicides [65]. Studies have demonstrated that herbicide contamination disrupts both nitrogen cycling and organic matter decomposition processes by suppressing key microbial populations [66,67,68], including *Limnobacter*.

Herbicide exposure can alter microbial community composition, which in turn may influence the distribution and abundance of ARGs [69]. In this study, we employed the network analysis to investigate the co-occurrence patterns between ARG types and microbial taxa. To enhance the reliability of our findings, we applied a stringent selection criterion, setting ρ > 0.9 as the correlation threshold alongside a significance level of *p* < 0.05. This threshold ensures the identification of only highly robust correlations, effectively minimizing spurious associations while improving the stability and biological interpretability of the network [70,71,72]. However, empirical evidence indicates that repeated applications of common herbicides can significantly increase the prevalence of ARGs and MGEs in soil bacteria, even in the absence of major shifts in the overall bacterial community composition [15]. Meanwhile, this may be due to herbicide stress inducing similar resistance mechanisms, such as multidrug efflux pumps or membrane modifications, across diverse microbial taxa [73]. Additionally, HGT via plasmids or integrons can decouple ARG distribution from specific hosts [74,75], making it difficult to detect clear taxon–ARG associations. Notably, four major ARGs (*ceoB*, *oprC*, *bpeF*, and *adeF*) exhibited similar distribution patterns (Figure 5), suggesting a potential co-enrichment within specific microbial communities. Interestingly, when the threshold for the network analysis was reduced (without considering the proportion of samples in which a gene was detected across all samples), a correlation emerged between the four resistance genes (*ceoB*, *oprC*, *bpeF*, *adeF*) and *sul1*. This suggests that the observed shifts in the abundance of these genes could be driven by HGT [76]. Additionally, it is plausible that, due to the inherent complexity of microbial communities—especially in herbicide-contaminated soils—ARGs may not be transferred through direct species interactions, but rather via other mechanisms, such as gene mutation and selective pressure [77]. Meanwhile, Blattabacterium, Gordonia, Providencia, and Brevibacterium were identified as hubs within their respective modules, suggesting that these microorganisms may play central roles in their specific microbial communities [78,79,80,81].

Our findings demonstrate the influence of herbicide contamination on microbial diversity and the distribution of ARGs, with important implications for agricultural sustainability, environmental stewardship, and public health. Meanwhile, the potential dissemination of ARGs through environmental pathways raises concerns regarding the acceleration of antibiotic resistance in both clinical and agricultural contexts [82,83]. Incorporating soil microbiome and ARG surveillance into public health monitoring frameworks could facilitate the early detection of resistance hotspots and inform targeted mitigation strategies.

While our study offers valuable insights into the effects of herbicide contamination on microbial diversity and ARG distribution, several limitations should be considered. First, reliance on publicly available metagenomic datasets may introduce biases due to variations in sampling [84]. Second, sample classification was based on metadata rather than direct chemical analyses, potentially affecting interpretations due to differences in herbicide concentrations and exposure histories [85]. Third, the limited sample size of control groups, particularly in Haikou, may have reduced statistical power [86]. Finally, in real environmental settings, it is often the metabolic byproducts of herbicides, rather than the parent compounds themselves, that exert significant effects on microbial community composition and the distribution of ARGs [87,88]. Therefore, this study did not prioritize the dose–response relationship of herbicide concentration. Additionally, the data were derived from publicly available third-party sources, where many potential systematic errors could not be controlled, limiting our ability to investigate the effects of herbicide levels in greater detail. Future studies should adopt standardized sampling, direct chemical measurements, and experimental validation to better understand the ecological impact of herbicide contamination on microbial communities and ARG dissemination.

## 5. Conclusions

This study highlights the significant ecological impact of herbicide contamination on microbial communities and the distribution of ARGs. The findings demonstrate that herbicides not only affect microbial diversity but also alter the abundance of ARGs, with the reduction in biodiversity in contaminated environments underscoring the toxic effects of herbicide exposure. Specifically, herbicide contamination was found to promote the spread of resistance genes, particularly multidrug, sulfonamide, and bacitracin resistance genes, with ARG subtypes such as *bacA*, *sul1*, and *novA* showing notable increases. The underlying mechanism is likely linked to herbicide-induced stress, which facilitates HGT. We also observed a correlation between *ceoB*, *oprC*, *bpeF*, and *adeF*. Further correlation analyses revealed a co-occurrence pattern between *ceoB*, *oprC*, *bpeF*, *adeF*, and *sul1*, suggesting that HGT plays a crucial role in the spread of antibiotic resistance in herbicide-contaminated soils. Microbial community analyses reveal a significant decrease in genera such as *Fibrisoma*, *Gilsonvirus*, *Limnobacter*, and *Wilnyevirus* under herbicide contamination. Environmental factors, such as geographic location, soil properties, and herbicide application intensity, were also shown to significantly influence microbial community composition and ARG distribution. This emphasizes the complexity and multifaceted nature of microbial resistance in agricultural ecosystems. In conclusion, this study provides valuable insights into the ecological changes driven by herbicide contamination and offers a foundation for assessing its long-term environmental and public health impacts.

## Figures and Tables

**Table 1:** Sample information for each group.

Group	Geographic Coordinates	Number of Samples	Sample Type	Type of Herbicide	Herbicide Contamination	Control Group?
S_Qingdao	36°4′33.4″ N, 120°24′30.7″ E	32	Soil	Chloroacetamide herbicides	Yes	No
CK_Qingdao	36°4′33.4″ N, 120°24′30.7″ E	4	Soil	\	No	Yes
S_Haikou	20°2′48.7″ N, 110°11′44.4″ E	9	Soil	Organophosphorous herbicides	Yes	No
CK_Haikou	20°2′48.7″ N, 110°11′44.4″ E	3	Soil	\	No	Yes
S_Nanjing	32°3′41.6″ N, 118°47′29.6″ E	35	Soil	Phenoxyalkanoic acid herbicides/Sulfonylurea herbicides/Systemic herbicides	Yes	No
S_Mianyang	31°27′45.8″ N, 104°44′46.8″ E	6	Soil	Systemic herbicides	Yes	No
S_Yingtan	28°20′37.8″ N, 116°55′45.5″ E	6	Soil	Systemic herbicides	Yes	No
S_Diamantina	18°11′12.9″ S, 43°32′14.6″ W	2	Soil	Triazine herbicides	Yes	No
W_Mont-Saint-Hilaire	45°32′ N, 73°09′ W	88	Water	Organophosphorous herbicides	Yes	No

## Data Availability

Data were collected from the National Center for Biotechnology Information Short Reads Archive database (NCBI SRA).

## Supplementary Materials

The following supporting information can be downloaded at: https://www.mdpi.com/article/10.3390/life15040547/s1, Supplementary Table S1: Information of tested datasets. Supplementary Table S2: Sample information for each group. Supplementary Figure S1: Changes in ARGs within different groups. Average of abundance in S_Nanjing (A), S_Yingtan (B), S_Mianyang (C), S_Haikou (D), S_Qingdao (E), S_Diamantina (F), W_Mont-Saint-Hilaire (G), CK_Qingdao (H), CK_Haikou (I). Supplementary Figure S2: Heatmap of average abundance of ARG types in different groups. Supplementary Figure S3: Heatmap of average abundance of shared ARGs across polluted soil groups (S_Nanjing, S_Yingtan, S_Mianyang, S_Haikou, S_Qingdao, S_Diamantina). Supplementary Figure S4: Pie chart of average abundance of bacterial communities at phylum level. Supplementary Figure S5: Pie chart of abundance of bacterial community at genus level in S_Nanjing and W_Mont-Saint-Hilaire. Supplementary Figure S6: Venn plots for number of ARGs subtypes in contaminated soil groups (S_Nanjing, S_Yingtan, S_Mianyang, S_Haikou, S_Qingdao, S_Diamantina) (A). Venn plots of ARGs subtypes in S_Qingdao and CK_Qingdao (B), S_Haikou and CK_Haikou (C). Supplementary Figure S7: Alpha diversity indices boxplot in different groups at species level (A). Principal coordinates analysis (PCoA) based on genus level between groups. X-axis, first principal component and Y-axis, second principal component; 36.465% in brackets represents contributions of PC1 components to samples, 12.766% represents contributions of PC2 components to samples. A dot represents each sample, and different colors represent different groups (B). Supplementary Figure S8: Volcano plot of differentially expressed ARGs in Qingdao and Haikou groups. Supplementary Figure S9. Linear regression analysis of bacterial community and ARG subtype diversity vs. total ARG abundance. Relationship between Shannon index of bacterial community at species level and total ARG abundance (R^2^ = 0.2966, *p* < 0.05) (a). Relationship between richness index of bacterial community at species level and total ARG abundance (R^2^ = 0.1541, *p* < 0.05) (b). Relationship between Shannon index of ARG subtypes and total ARG abundance (R^2^ = 0.01684, *p* > 0.05) (c). Relationship between richness index of ARG subtypes and total ARG abundance (R^2^ = 0.458, *p* < 0.05) (d). Supplementary Figure S10. Linear regression analysis of bacterial community vs. total ARG abundance. Relationship between Shannon index of bacterial community and total ARG abundance (R^2^ = 0.1339, *p* < 0.05) (a). Relationship between richness index of bacterial community and total ARG abundance (R^2^ = 0.3535, *p* < 0.05) (b).

## Abbreviations

ARGs	antibiotic resistance genes
MGEs	mobile genetic elements
HGT	horizontal gene transfer
PCoA	principal coordinates analysis
